# SKIN COLOR DIFFERENCES AND HEALTH-RELATED QUALITY OF LIFE PERCEPTION AFTER GASTRIC BYPASS ROUX-EN-Y: A CROSS-SECTIONAL STUDY

**DOI:** 10.1590/0102-6720202500003e1872

**Published:** 2025-03-03

**Authors:** Milca Rodrigues Vieira de ANDRADE, Ana Rafaela Soares do VALE, Mariana Sousa de Pina SILVA, João Henrique Cerqueira BARROS, Laura Souza LAGARES, Luiz Alberto Bastos de ALMEIDA, Carolina Villa Nova AGUIAR, Clarcson Plácido Conceição dos SANTOS

**Affiliations:** 1 Escola Bahiana de Medicina e Saúde Pública, Research Group on Metabolic Diseases Physical Exercise and Health Technologies - Salvador (BA), Brasil; 2 Escola Bahiana de Medicina e Saúde Pública, Professional Master’s Program in Health Technologies - Salvador (BA), Brasil; 3 Escola Bahiana de Medicina e Saúde Pública, Postgraduate Program in Medicine and Human Health - Salvador (BA), Brasil; 4 Universidade de Feira de Santana, Physical Activity Laboratory State - Feira de Santana (BA), Brasil

**Keywords:** Ethnicity, Quality of Life, Bariatric Surgery, Etnicidade, Qualidade de Vida, Cirurgia Bariátrica

## Abstract

**BACKGROUND:**

Differences in skin color have socioeconomic and health implications; however, gaps persist in understanding health-related quality of life (HRQoL) perception.

**AIMS:**

To examine whether skin color differences influence HRQoL in obese patients undergoing Roux-en-Y gastric bypass surgery.

**METHODS:**

Cross-sectional study with participants of both genders, aged 18 to 60, and three to six months postoperatively. Data were collected from October 2018 to July 2019 at a bariatric clinic in Salvador, Bahia. Skin color, Moorehead-Ardelt II Quality of Life Questionnaire (MAQOL-II) scores, anthropometric measurements, socioeconomic status, physical activity, and body image perceptions were recorded.

**RESULTS:**

Of 196 patients, 67.35% were Black. “Self-esteem” in MAQOL-II demonstrated the most significant post-surgical improvement, with 62.8% reporting “much better” outcomes. Adjusted residuals associated “much better” and “unchanged” responses with skin color. The overall MAQOL-II score indicated lower HRQoL scores (M=1.65; standard deviation - SD=0.98) for individuals with black skin compared to those with white skin. Analyzing questionnaire responses, both racial groups exhibited equal percentages (45.3%) reporting “much better” and “better” post-surgery progress. However, no statistically significant differences in HRQoL were observed when comparing skin color.

**CONCLUSIONS:**

Skin color appears not to significantly impact the HRQoL of obese patients undergoing Roux-en-Y gastric bypass.

## RESUMO

**RACIONAL:**

Diferenças na cor da pele têm implicações socioeconômicas e de saúde, no entanto, ainda existem lacunas na compreensão da percepção da qualidade de vida relacionada à saúde (HRQoL).

**OBJETIVOS:**

Examinar se as diferenças na cor da pele influenciam a HRQoL em pacientes com obesidade submetidos à cirurgia de bypass gástrico Roux-en-Y.

**MÉTODOS:**

Estudo transversal com participantes de ambos os sexos, idades entre 18 e 60 anos e 3 a 6 meses de pós-operatório. Os dados foram coletados de outubro de 2018 a julho de 2019 em uma clínica bariátrica em Salvador, Bahia. A cor da pele de cada participante, escores do Questionário de Qualidade de Vida Moorehead-Ardelt II (MAQOL-II), medidas antropométricas, status socioeconômico, atividade física e percepções da imagem corporal foram registrados.

**RESULTADOS:**

Dos 196 pacientes, 67,35% eram negros. A “Autoestima” no MAQOL-II demonstrou a melhoria pós-cirúrgica mais significativa, com 62,8% relatando resultados “Muito melhores”. Resíduos ajustados associaram respostas “Muito melhores” e “Inalteradas” com a cor da pele. O escore geral do MAQOL-II indicou escores de HRQoL mais baixos (M=1,65; DP=0,98) para indivíduos com pele negra em comparação com aqueles com pele branca. Ao analisar as respostas do questionário, ambos os grupos raciais apresentaram percentuais iguais (45,3%) relatando progresso “Muito melhor” e “Melhor” pós-cirúrgico. No entanto, não foram observadas diferenças estatisticamente significativas na HRQoL ao comparar a cor da pele.

**CONCLUSÕES:**

A cor da pele parece não impactar significativamente na HRQoL de pacientes com obesidade submetidos ao bypass gástrico Roux-en-Y.

## INTRODUCTION

Obesity is considered one of the main public health problems according to the World Health Organization (WHO)^28^ ^,^ ^33^. This condition generates several health problems for the individual, not only because it increases the risk factor for numerous metabolic diseases, but also for mental health and quality of life^22^ ^,^ ^28^. Thus, the treatment of obesity is essential for an overall improvement of individuals affected by this condition. Bariatric surgery was demonstrated to be one of the most effective therapies in weight loss and in the improvement of health-related quality of life (HRQoL) of patients with obesity, even when directly compared with other therapies^3^ ^,^ ^13^ ^,^ ^17^ ^,^ ^30^.

However, it is worth mentioning that Brazil is a country that shows several racial, cultural, and social disparities^20^. Thus, a Brazilian living with obesity who seeks bariatric surgery may be faced with a multitude of factors that directly influence the chosen treatment for obesity^21^. The Brazilian healthcare resources available often exhibit disparities across different racial groups due to historical and socioeconomic factors^5^ ^,^ ^9^. Notably, within Brazil, individuals of Black ethnicity have frequently been associated with lower socioeconomic status, facing increased challenges in accessing quality healthcare services^10^.

Race and ethnicity are two different concepts; while race encompasses phenotypic characteristics such as skin color, hair type, and facial and cranial conformation, ethnicity comprises cultural factors surrounding nationality, religion, and traditions, among others. Thus, as this article deals with the issue of quality of life correlated to the specific skin color of the participants, the term race will be adopted to refer to the research participants^11^.

Nevertheless, the issue of obesity introduces a complex dimension to this scenario. While economic status plays a role in shaping health outcomes, obesity and HRQoL emerge not only from access to healthcare resources, but from a combination of factors, including genetics, predisposition through epigenetic mechanisms such as environment, lifestyle choices, and psychological well-being^25^. Therefore, the need emerges to understand the impacts of these complex biological, economic, and psychological dimensions in the post-operative outcomes of obesity treatment, especially in terms of HRQoL^6^. However, despite numerous studies on post-operative outcomes, little is known about the influence of skin color on the HRQoL improvements experienced by individuals of diverse racial backgrounds after this intervention. 

Thus, this study aims to determine whether skin color influences HRQoL among obese people undergoing Roux-en-Y gastric bypass.

## METHODS

### Study design and sample

This study adopted a cross-sectional design to collect data from adult volunteers of both sexes, aged between 18 and 60 years, during the period from October 2018 to July 2019. The sample was selected by convenience, and information regarding age, skin color, body mass, and height was collected at a regional reference private clinic specializing in bariatric surgery in the city of Salvador, Bahia. Participants had to be within the three to six months period post-bariatric surgery. Individuals with cognitive impairment and/or who were illiterate were not invited to take part in the study.

### Moorehead-Ardelt II Quality-of-Life Questionnaire

The Moorehead-Ardelt II Quality-of-Life Questionnaire (MAQOL-II) questionnaire is a validated tool used to assess the impact of bariatric surgery on individuals’ perception of HRQoL post-procedure^6^. Developed from the Bariatric Analysis and Reporting System (BAROS)^16^, the questionnaire employs a scale of figures representing six domains:

1. Self-esteem;
2. Physical activities;
3. Social contacts;
4. Satisfaction with work performance;
5. Interest in affection and/or sex; and
6. Eating habits.

For this study, the shorter version of the questionnaire was utilized, which includes the first five domains in the analysis. Each domain has a scale ranging from -0.50 to +0.50, and their final scores contribute to an HRQoL score with values between -3 and +3. These scores are categorized as follows: -3 to -2.1: very bad; -2 to -1.1: bad; -1 to 1: fair; 1.1 to 2: good; 2.1 to 3: very good^16^.

### Anthropometric measurements

Anthropometric measurements, encompassing body mass and height, were obtained through electronic medical records, as these measurements are consistently taken in a standardized manner by the clinic’s multidisciplinary team and recorded in the medical records. Trained observers, qualified for data collection, recorded the objective measurements, and compared them to the scale of figures used in the study.

### Socioeconomic questionnaire

The Brazilian Economic Classification Criteria (CCEB) were utilized to address socioeconomic aspects, constituting a classification system for the Brazilian population based on the ownership of assets, rather than assessing social classes solely on family income. Each owned asset corresponds to a specific score, and the total score defines an individual’s socioeconomic class. For this research, the CCEB 2012 version, incorporating data from the socioeconomic survey conducted by the Brazilian Association of Research Companies (ABEP) in 2010, was employed. Socioeconomic classes are delineated by the CCEB, considering family income in Brazilian Real (BRL). The classification includes A1 (BRL 12,926), A2 (BRL 8,418), B1 (BRL 4,418), B2 (BRL 2,565), C1 (BRL 1,541), C2 (BRL 1,024), D (BRL 714), and E (BRL 477). The survey respondents were required to provide information about asset possession, family income, and level of education/education of the family head^4^.

### International Physical Activity Questionnaire

The International Physical Activity Questionnaire (IPAQ) is a globally recognized and validated survey designed to assess the weekly time spent on moderate and vigorous physical activities across various domains, including work, transportation, housework, and leisure. Additionally, it measures time spent in sedentary activities performed while seated^19^. For this study, the short version of the questionnaire was utilized. To determine whether individuals are physically active, they must meet the minimum requirements recommended by the WHO for weekly physical activity. This includes engaging in vigorous-intensity activities for at least three days per week, with each session lasting at least 20 minutes, or engaging in moderate-intensity activities or walking for at least five days per week, with each session lasting at least 30 minutes. Alternatively, individuals can achieve the recommended activity level by combining various activities over a week, totaling at least 150 minutes per week^19^.

### Stunkard’s image rating scale

The silhouette scale developed by Stunkard et al. is designed to subjectively assess individuals’ perceptions of their body size and shape. The method employs a series of figures that objectively varies from the thinnest to the heaviest, and participants are asked to select the figure that best represents their current, ideal, or desired body shape. The scale consists of nine Stunkard figures^27^.

By comparing the subjective body mass index (BMI) obtained through the silhouette scale with the BMI derived from objective measurements of body mass and height, the researchers established a correlation^29^. Using the questionnaire, the difference between the scores for the real and ideal body images was calculated. A score of zero indicates that the volunteer is satisfied with their body image, a positive score suggests dissatisfaction due to excess weight, and a negative score indicates dissatisfaction due to thinness^27^. This method allows for understanding participants’ perceptions of their body image and their level of satisfaction with it.

### Statistical analysis

To test the normality of continuous variables, descriptive data analysis and the Shapiro-Wilk test were used. We then performed a descriptive analysis of the data, providing measures of central tendency, such as mean, and measures of dispersion, such as standard deviation. In addition, absolute and relative values were determined based on the categorical variables, and, for comparison of these measures, the chi-square test was used. Subsequently, we compared the descriptive data and the MAQOL-II instrument scores between the groups according to skin color, considering the Black and White groups. This comparison allowed us to identify possible differences in HRQoL between groups based on skin color. To compare the MAQOL-II quantitative scores considering skin color, we used Student’s t-test for independent reflection to investigate whether there are differences that affect HRQoL scores between groups. For the analysis of the MAQOL-II scores, the chi-square test with modified residues was performed and to evaluate the magnitude of effect, the Cohen’s test was used. This test allowed us to examine whether there is a statistically significant association between skin color and HRQoL scores. In addition, we performed a frequency analysis stratified by groups, grouping individuals according to the MAQOL-II results. These analyses allowed us to explore possible associations between HRQoL scores and skin color. Finally, for statistical analysis, we adopted a significance level of 5%.

This research project obtained approval from the Ethics in Research Committee of the Bahian School of Medicine and Public Health (EBMSP) (number 2.881.471, on September 9, 2018). The study was conducted in adherence to Resolution No. 466/12 issued by the National Health Council. The information gathered was used solely for this survey, ensuring strict data confidentiality and the anonymity of all participants.

## RESULTS

A total of 196 individuals were included as participants in this study, with mean ages of 39.9 ± 9.8 years for blacks and 41.3±10.7 years for whites, and the female prevalence was 80.3 and 68.8% respectively. The racial distribution of patients was 132 (67.35%) Black and 64 (32.65%) White. Blacks were heavier than Whites (BMI 32.2 vs. 31.1 kg/m^2^) and more physically inactive (53 vs. 43 %, respectively). Additionally, most individuals had a similar socioeconomic profile, with 56.1% of Black individuals and 75% of White individuals classified as class B and dissatisfaction with being overweight was reported by 96.2% of Blacks and 89.1% of Whites. Table 1 describes the characteristics of individuals after bariatric surgery. None of the analyzed variables demonstrated statistical significance.

**Table 1:** Sample characteristics.

Variables	All patients	Black	White	p-value
	n=196	n=132	n=64	
Age (years)	40.4 (10.1)	39.9 (9.8)	41.3 (10.7)	0.37
Weight (kg)	87.8 (15.4)	88.1 (14.5)	87.3 (17)	0.76
BMI (kg/m^2^)	32 (4.6)	32.2 (4.4)	31.1 (4.7)	0.12
Surgery time (months)	4.4 (1.3)	4.3 (1.3)	4.4 (1.4)	0.66
Sex n (%)				
Women	150 (76.5)	106 (80.3)	44 (68.8)	0.74
Men	46 (23.5)	26 (19.7)	20 (31.3)	
Marital status n (%)				
Single	69 (35.2)	46 (34.8)	23 (35.9)	0.66
Married	96 (49)	67 (50.8)	29 (45.3)	
Divorced	13 (6.6)	9 (6.8)	4 (6.3)	
Widower	1 (0.5)	1 (0.8)	0	
Stable union	17 (8.7)	9 (6.8)	8 (12.5)	
Education n (%)				
Complete Basic Ed.	2 (1)	1 (0.8)	1 (1.6)	0.35
Incomplete Basic Ed.	3 (1.5)	1 (0.8)	2 (3.1)	
High School	35 (17.9)	29 (22)	6 (9.4)	
Incomplete High School	4 (2)	3 (2.3)	1 (1.6)	
Incomplete Higher Ed.	62 (31.6)	41 (31.1)	21 (32.8)	
Bachelor	30 (15.3)	18 (13.6)	12 (18.8)	
Graduate	60 (30.6)	39 (29.5)	21 (32.8)	
Socioeconomic profile n (%)				
Class A	17 (8.7)	14 (10.6)	3 (4.7)	0.06
Class B	122 (62.2)	74 (56.1)	48 (75)	
Class C	57 (29.1)	44 (33.3)	13 (20.3)	
Physical activity profile n (%)				
Physically inactive	98 (50)	70 (53)	28 (43.8)	0.22
Physically active	98 (50)	62 (47)	36 (56.3)	
Body satisfaction n (%)				
Satisfied	11 (5.6)	5 (3.8)	6 (9.4)	0.09
Dissatisfied with thinness	1 (0.5)	0	1 (1.6)	
Dissatisfied with being overweight	184 (93.9)	127 (96.2)	57 (89.1)	

BMI: body mass index.

The variables age, weight, BMI, and surgery time are presented as mean (standard deviation), and Student’s t-test for independent samples was used to determine the p-value. Categorical variables are presented as frequency (proportion) and the chi-square test was used to determine the p-value.

Applying the MAQOL-II, “self-esteem” was the greatest outcome upgraded after surgery, with 123 (62.8%) patients expressing a “much better” improvement in this domain, with 80 (60.6%) of this group being White and 43 (67.2%) Black. When it comes to “willingness for physical activity”, “willingness for social relationships” and “willingness to work”, a “much better” improvement was found in most of the Black group (respectively, 54.7; 50; 46.9%) and only a “better” development for most of the White patients (respectively, 51.5; 38.6; 42.4%). In terms of “sexual interest”, none of the groups analyzed had a prevalence of a “much better” improvement in this domain. A group of 25 (39.1%) Black patients had a “better” development and 50 (37.9%) White individuals had no alteration after the bariatric surgery in this category. Table 2 describes the answers to this questionnaire.

**Table 2:** Results of data from the Moorehead-Ardelt Quality of Life Questionnaire II (MAQOL-II) analyzed by domain and stratified by skin color.

Domains	All patients	Black	White	p-value
	n=196	n=132	n=64	
Self-esteem n (%)				
Much better	123 (62.8)	80 (60.6)	43 (67.2)	0.74
Better	70 (35.7)	49 (37.1)	21 (32.8)	
Unaltered	1 (0.5)	1 (0.8)	0	
Worse	1 (0.5)	1 (0.8)	0	
Much worse	1 (0.5)	1 (0.8)	0	
Willingness for physical activity n (%)				
Much better	89 (45.4)	54 (40.9)	35 (54.7)	0.36
Better	94 (48)	68 (51.5)	26 (40.6)	
Unaltered	10 (5.1)	8 (6.1)	2 (3.1)	
Worse	2 (1)	1 (0.8)	1 (1.6)	
Much worse	1 (0.5)	1 (0.8)	0	
Willingness for social relationship n (%)				
Much better	81 (41.3)	49 (37.1)	32 (50)	0.38
Better	70 (35.7)	51 (38.6)	19 (29.7)	
Unaltered	40 (20.4)	29 (22)	11 (17.2)	
Worse	4 (2)	2 (1.5)	2 (3.1)	
Much worse	1 (0.8)	1 (0.8)	0	
Willingness to work n (%)				
Much better	84 (42.9)	54 (40.9)	30 (46.9)	0.67
Better	78 (39.8)	56 (42.4)	22 (34.4)	
Unaltered	32 (16.3)	20 (15.2)	12 (18.8)	
Worse	1 (0.5)	1 (0.8)	0	
Much worse	1 (0.5)	1 (0.8)	0	
Sexual interest n (%)				
Much better	51 (26)	3 (2.3)	18 (28.1)	0.2
Better	62 (31.6)	37 (28)	25 (39.1)	
Unaltered	64 (32.7)	50 (37.9)	14 (21.9)	
Worse	13 (6.6)	9 (6.8)	4 (6.3)	
Much worse	6 (3.1)	3 (2.3)	3 (4.7)	

The data is presented as frequency (proportion) and the χ^2^ test was used to determine the p-value.

Moreover, Table 3 indicated a measure used to assess the strength and direction of these associations. The association was calculated based on the observed and expected frequencies, and how each race contributes to the association between the variables. A positive value indicates a stronger association, while values close to zero suggest that there is no strong or evident association between the variables. The results of our analysis, based on the MAQOL-II questionnaire responses, indicated potential associations between the variables “much better” and “unchanged” and skin color. However, these associations did not reach statistical significance (p>0.05), indicating that the observed results may have occurred randomly.

**Table 3:** Analysis of Moorehead-Ardelt Quality of Life questionnaire II scores (MAQOL-II), with residuals adjusted and stratified by skin color.

Skin color	Scores MAQOL-II			
	Much better	Better	Unaltered	Worse
Black (n)	43	65	21	3
Adjusted residuals	1.7*	0.5*	1.9*	-0.4*
White (n)	29	29	4	2
Adjusted residuals	-1.7*	-0.5*	-1.9*	0.4*

n: sample; 4x2 independence χ^2^ test. *p>0.05.

**Figure 1:**
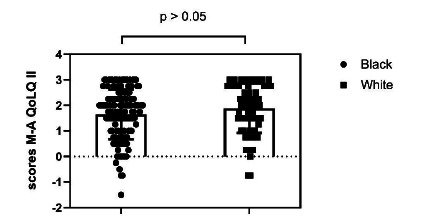
Moorehead-Ardelt Quality of Life questionnaire II overall score, stratified by skin color.

The overall score of MAQOL-II showed that people with black skin had lower HRQoL scores (M=1.65; SD = 0.98) when compared to people with white skin (M=1.89; SD=0.96), but with no statistically significant difference (t [194]=1.602, p>0.05) (Figure 1). Furthermore, the effect size of the difference was low (Cohen’s d=0.24).

**Figure 2:**
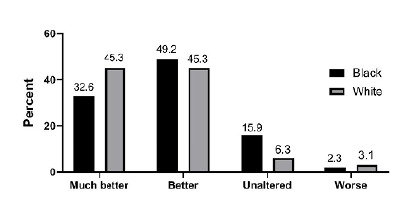
Overall distribution by groups of the results of the Moorehead-Ardelt Quality of Life Questionnaire II, stratified by skin color.

When distributed by frequency of answers in the questionnaire, the white group had equal 45.3% “much better” and “better” development after bariatric surgery. Most Black patients (49.2%) reported a “better” improvement since the surgery. Figure 2 shows the frequencies of answers in both groups.

## DISCUSSION

This study examined HRQoL in patients of variable skin colors who experienced bariatric surgery. Even with an improvement in MAQOL-II questionnaire results in both groups, our findings had no significant statistical differences when stratifying those answers by race, suggesting that skin color does not correspond to a predictable factor for better or worse results in quality of life after metabolic surgery. 

The study’s results showed a notable enhancement in quality of life for both the Black and White populations, with individuals from both racial backgrounds experiencing positive changes in this aspect. While differences were observed in specific questionnaire domains between the groups, it is encouraging to note that both populations experienced comparable and significant improvements in overall quality of life. These findings are highly consistent with previously conducted systematic reviews, which have demonstrated that bariatric surgery is associated with improved quality of life across diverse racial groups^3^ ^,^ ^15^ ^,^ ^17^ ^,^ ^23^.

In addition, the analysis of recent studies associated the highest level of HRQoL with the percentage of weight loss and with a peak of those levels occurring in the first year after surgery^13^. These results also contribute to our findings, since our study was conducted at the same time that the peak score should happen. Therefore, there must be a concern with the weight variability of those patients in their immediate and long-term follow-up, due to its direct connection with HRQoL.

However, all the analyses conducted in this study indicated a lack of statistically significant variation in the level of improvement in quality of life among individuals from both racial backgrounds. This finding contrasts with a previous study, which revealed that Black patients were less likely to report “good” or “very good” quality of life one year after surgery^32^. Nevertheless, it is worth noting that the questionnaire utilized by the researchers in that study was not validated specifically for evaluating the studied population, which may have influenced the results and could explain the contrasting findings.

There are just a few studies in the literature comparing post-operative outcomes of bariatric surgery for different skin colors. In those results, Black patients have higher odds of presenting complications after surgery. These higher chances of presenting post-surgical complications are due to the fact that Brown and Black races have unequal access and, consequently, greater difficulty in accessing healthcare in Brazil^23^. However, none of these studies presented an analysis of HRQoL using any of the questionnaires available nowadays^1^ ^,^ ^2^ ^,^ ^8^ ^,^ ^31^. Therefore, it is impossible to determine a relationship between such findings and quality of life, with the need for more detailed studies in the future. 

Furthermore and noteworthy, bariatric surgery was not expected to be as effective in improving the quality of life in Black people precisely because, in a pathological context, all aspects that make up a society must be considered when assessing quality of life^7^. It was expected that the more vulnerable the individual, especially in the sense of being subjected to racism in society, the greater the social pressure on them^24^. However, obesity is a multifactorial disease, arising from a lack of information and access to health services, in addition to environmental and genetic factors, thus becoming a universal disease that affects all ethnic groups^26^. Therefore, as evidenced by this study, bariatric surgery equally influences the quality of post-surgical life regardless of race.

As mentioned before, weight loss has a significant impact on quality of life^13^. A study conducted by researchers revealed some factors that can influence weight variability in the Black population, such as social and economic status, cultural dietary practices, ethnic differences in the perception of desired weight and disparities in access to medical and follow-up care^18^. Therefore, understanding those socioeconomic, psychological, and medical differences could potentially optimize aspects that would negatively impact weight loss and, consequently, quality of life in the course of bariatric surgery in this group. 

Moreover, the scientific literature supports the positive impacts of surgical intervention in improving the quality of life in patients with obesity^12^ ^,^ ^14^ ^,^ ^30^. Therefore, these findings suggest that bariatric surgery can be a beneficial option for enhancing quality of life, regardless of skin color.

Our findings must be interpreted in the context of the study’s limitations. The inclusion of participants exclusively from a private clinic in our investigation may limit the generalization of our findings to individuals undergoing bariatric procedures in different healthcare settings. In addition, uncontrolled variables, such as participants’ medical history, including pre-existing chronic health conditions, and the use of medications, can also play a role in the observed results. Lastly, the absence of standardized instruments in the literature for evaluating quality of life in individuals after bariatric surgery poses challenges when it comes to comparing data.

## CONCLUSIONS

Findings from our study suggest that there is no substantial evidence to support the notion that skin color plays a noticeable role in impacting HRQoL outcomes of individuals undergoing Roux-en-Y gastric bypass surgery. This conclusion emphasizes the importance of addressing health-related disparities through a broader perspective than skin color alone.

## Appendix

Central Message Bariatric surgery was demonstrated to be one of the most effective therapies in weight loss and in the improvement of health-related quality of life (HRQoL) of patients with obesity, even when directly compared with other therapies. Brazil is a country that shows several racial, cultural, and social disparities. Thus, a Brazilian living with obesity who seeks bariatric surgery may be faced with a multitude of factors that directly influence the chosen treatment for obesity. Race and ethnicity are two different concepts. While race encompasses phenotypic characteristics such as skin color, hair type, and facial and cranial conformation, ethnicity comprises cultural factors surrounding nationality, religion, and traditions, among others.

Perspectives The scientific literature supports the positive impacts of surgical intervention in improving the quality of life in patients with obesity. Therefore, our findings suggest that bariatric surgery can be a beneficial option for enhancing quality of life, regardless of skin color. The findings from this study suggest that there is no substantial evidence to support the notion that skin color plays a noticeable role in impacting the health-related quality of life (HRQoL) outcomes of individuals undergoing Roux-en-Y gastric bypass surgery.
